# A cross-sectional study of pre-travel health-seeking practices among travelers departing Sydney and Bangkok airports

**DOI:** 10.1186/1471-2458-12-321

**Published:** 2012-05-02

**Authors:** Anita E Heywood, Rochelle E Watkins, Sopon Iamsirithaworn, Kessarawan Nilvarangkul, C Raina MacIntyre

**Affiliations:** 1 School of Public Health and Community Medicine, Faculty of Medicine, University of New South Wales, Kensington, NSW, 2052, Australia; 2 Telethon Institute of Child Health Research, Centre for Child Health Research, The University of Western Australia, Perth, Australia; 3 Bureau of Epidemiology, Department of Disease Control, Ministry of Public Health, Bangkok, Thailand; 4 Research and Training Center for Enhancing Quality of Life of Working-Age People, Faculty of Nursing, Khon Kaen University, Khon Kaen, Thailand; 5 National Centre for Immunisation Research and Surveillance of Vaccine Preventable Diseases (NCIRS), The Children’s Hospital at Westmead and Discipline of Paediatrics and Child Health, University of Sydney, New South Wales, Australia

## Abstract

****Background**:**

Pre-travel health assessments aim to promote risk reduction through preventive measures and safe behavior, including ensuring travelers are up-to-date with their immunizations. However, studies assessing pre-travel health-seeking practices from a variety of medical and non-medical sources and vaccine uptake prior to travel to both developing and developed countries within the Asia-Pacific region are scarce.

****Methods**:**

Cross-sectional surveys were conducted between July and December 2007 to assess pre-travel health seeking practices, including advice from health professionals, health information from other sources and vaccine uptake, in a sample of travelers departing Sydney and Bangkok airports. A two-stage cluster sampling technique was used to ensure representativeness of travelers and travel destinations. Pre-travel health seeking practices were assessed using a self-administered questionnaire distributed at the check-in queues of departing flights. Logistic regression models were used to identify significant factors associated with seeking pre-travel health advice from a health professional, reported separately for Australian residents, residents of other Western countries and residents of countries in Asia.

****Results**:**

A total of 843 surveys were included in the final sample (Sydney 729, response rate 56%; Bangkok 114, response rate 60%). Overall, pre-travel health information from any source was sought by 415 (49%) respondents with 298 (35%) seeking pre-travel advice from a health professional, the majority through general practice. Receipt of a pre-travel vaccine was reported by 100 (12%) respondents. Significant factors associated with seeking pre-travel health advice from a health professional differed by region of residence. Asian travelers were less likely to report seeking pre-travel health advice and uptake of pre-travel vaccines than Australian or other Western travelers. Migrant Australians were less likely to report seeking pre-travel health advice than Australian-born travelers.

****Conclusions**:**

This study highlights differences in health-seeking practices including the uptake of pre-travel health advice by region of residence and country of birth. There is a public health need to identify strategies targeting these travel groups. This includes the promotion of affordable and accessible travel clinics in low resource countries as traveler numbers increase and travel health promotion targeting migrant groups in high resource countries. General practitioners should play a central role. Determining the most appropriate strategies for increasing pre-travel health preparation, particularly for vaccine preventable diseases in travelers is the next stage in advancing travel medicine research.

## **Background**

International travel has increased dramatically over the last few decades, in magnitude, speed and geographical reach with 940 million arrivals reported in 2010 [1]. Furthermore, the last decade has seen an increase in travel in the Asia-Pacific region well above the global average, resulting in a 21% share of international arrivals in 2010, up from 16% in 2000 [1]. Travelers play a significant role in the spread of infectious diseases across international borders, through their travel patterns and behaviors. Travel maybe the only risk factor for infectious diseases that are well controlled in the travelers’ country of residence, particularly vaccine-preventable diseases such as hepatitis A, typhoid, polio and measles. The role of vaccination among travelers is an essential component of national control of travel-associated infectious diseases. Understanding the behaviors of travelers and their attitudes towards a variety of communicable diseases can inform policy aimed at protecting the individual traveler, their contacts and the communities into which they travel. Behavioral studies of travelers provide insights into risks of both acquiring and importing infectious diseases.

A pre-travel consultation with a health professional can provide the international traveler with the necessary preventive advice on minimizing health risks during travel, including the risk of infectious disease and the opportunity for relevant vaccination and chemoprophylaxis. Travelers who seek pre-travel health advice from a health professional have been found to have better knowledge of infectious disease risk, more accurate risk perceptions and a higher level of intended risk-reducing behaviors [2-5]. Yet studies have shown that many Western travelers do not consult a health professional prior to travel and may not be aware of their need to protect themselves from infectious diseases [6-10]. Studies on the pre-travel health seeking behavior of travelers within the Asia-Pacific are limited; however, recent evidence suggests that the proportion of Asian travelers seeking pre-travel health advice is significantly lower than that of Western travelers [10]. Our study aimed to identify health-seeking practices among travelers and assess the proportion of travelers who sought pre-travel health advice and the uptake of pre-travel vaccines in a representative sample of travelers departing Sydney and Bangkok airports. The study also aimed to identify significant factors associated with pre-travel health seeking from a health professional.

## **Methods**

Cross-sectional surveys of travelers were conducted prior to their departure from international airports in Sydney, Australia for destinations in Asia between July and September 2007 and Bangkok, Thailand bound for Australia between October and December 2007. Asian destinations were defined according to the United Nations World Macro classification of regions and included countries in Eastern Asia and South-Eastern Asia [11]. Asian destinations were selected due to their close proximity to Australia and the high proportion of air traffic flow between these regions. The methods used in this study have been published previously [12]. Briefly, a two-stage cluster sampling technique was developed at each study site to randomly sample travelers. At Sydney airport, in the first stage, sample sizes for each destination were calculated based on the proportion of passengers departing for the selected destinations, derived from aviation statistics for the previous calendar year [13]. Total sample size was sufficient to estimate a population proportion of 50% with ±3.5% precision at the 5% confidence level. A representative sample of direct flights on all available days and times of departure for each pre-approved airline carrier for each of the selected destinations were included in an interviewing timetable. Airline carriers representing both Australian and non-Australian carriers were selected based on their total share of passengers with 11 of the 13 approached airlines providing approval.

The second stage of the cluster sampling method involved the distribution of questionnaires to every fifth passenger joining check-in queues of selected flights. Bilingual interviewers attended check-in counters 3 h before scheduled departure until 1 h before departure. A similar method was employed at the Bangkok airport, with selected flights proportionate to the number of traveler arrivals at Australian airports from Thailand and representative of Thai, Australian, and other carriers. Approximately 175 flights were sampled between July and September 2007 at the Sydney site comprising 2.7% of the flights to Asia during this period, and 13 flights between October and December 2007 at the Bangkok site, comprising 2.4% of the flights to Australia from Thailand during this period.

Eligible respondents were both visitors and residents aged 18 years or older, departing on the day of interview, excluding passengers in transit. The questionnaire was developed in simplified English and piloted at Sydney Airport. The revised questionnaire was translated into Chinese, Vietnamese and Thai with back-translation employed to ensure consistent interpretation of questions. The self-administered questionnaire included questions on socio-demographic characteristics and travel characteristics of their current trip. Respondents were provided with a list of health professionals and non-medical sources of travel health information. Pre-travel health seeking practices refer to both the seeking of health advice from a health professional as well as health information from other sources. Pre-travel health advice refers only to information obtained from a health professional. A list of common routine and travel vaccines were provided to respondents, who were asked to record vaccines received for the specific purpose of their current trip.

The sample obtained at the Sydney study site was weighted by flight destination to reflect the proportion of passenger departures from aviation statistics [13], due to over sampling of some destinations, thereby providing a representative sample of travelers departing Australia for destinations in Asia and avoiding sampling bias. No weighting was applied to the Bangkok sample. Data were analyzed using SPSS version 18 (SPSS Inc., Chicago, Illinois) and missing data were excluded from analysis. Statistical significance in bivariate analysis was assessed using the chi-squared test and we considered a p-value of <0.05 to be significant. Logistic regression analysis was undertaken to determine associations between socio-demographic and travel characteristics and the reported uptake of pre-travel health advice from a health professional. All variables which may plausibly predict uptake of pre-travel health advice with a significance of <0.25 on bivariate analysis were considered for inclusion in the logistic regression analysis [14], and final models were assessed for adequacy of sample sizes [15]. Variables considered plausible include: demographics; age, gender, marital status, education, employment, resident of birth country, and trip characteristics; length of stay, reason for travel, travel companions, destination sub-region, number of destination countries, within country air and train travel and attendance at crowded events. During multivariate logistic regression model fitting, significant independent variables associated with seeking pre-travel health advice differed for Australian travelers, other Western travelers and Asian travelers and separate models were developed. Travelers from other regions were too few to model. The proportion reporting uptake of pre-travel vaccination was insufficient to determine a valid logistic regression model and is reported as univariate analysis. The research was approved by the Human Research Ethics Committees of the University of Sydney, Australia (12–2006/9727), the University of New South Wales, Australia (08254), the Ministry of Public Health, Thailand (3–2399-00051–49-4) and the relevant airport authorities.

## **Results**

### **Study sample and travel profile**

A total of 843 surveys, including 729 weighted surveys of travelers departing Sydney to destinations in Asia (response rate; 56%), and 114 surveys of travelers departing Bangkok (response rate 60.0%) were included in the final sample. The number of respondents by flight destination has been reported previously [12]. Of the Sydney sample, 329/729 (45.1%) were residents of Australia, 170 (23.3%) were residents of other Western countries including New Zealand and countries in North America and Europe and 211 (28.9%) were residents of countries in Asia. Of the Bangkok sample, 55/114 (48.2%) were residents of Australia, 29 (25.4%) were residents of other Western countries and 14 (12.3%) were residents of countries in Asia. The demographic and travel characteristics, including travel activities by region of residence are shown in Table 1.

**Table 1:** **Traveler and trip characteristics by traveler region of residence** (N = 808*)

	**Residents of:**			
	**Australia** (N = 384)	**Other Western countries**^†^ (N = 199)	**Asia** (N = 225)	***p*-value**
**Demographics**				
**Age**				
16–35 years	185 (48.2%)	144 (72.4%)	133 (59.1%)	<0.001
36–55 years	124 (32.3%)	29 (14.6%)	69 (30.7%)	
>55 years	74 (19.3%)	26 (13.1%)	23 (10.2%)	
**Female**	165 (43.0%)	106 (53.3%)	111 (49.3%)	0.05
**Married**	206 (53.6%)	53 (26.6%)	107 (47.6%)	<0.001
**Resident of country of birth**	212 (55.2%)	175 (87.9%)	200 (88.9%)	<0.001
**Education**				
≤High school	78 (20.3%)	41 (20.6%)	44 (19.6%)	0.01
Tertiary trade/diploma	117 (30.5%)	51 (25.6%)	39 (17.3%)	
≥University bachelor degree	181 (47.1%)	104 (52.3%)	138 (61.3%)	
**Student**	38 (9.9%)	56 (28.1%)	81 (36.0%)	<0.001
**Trip characteristics**				
**Purpose of travel**				
Holiday	170 (44.3%)	118 (59.3%)	58 (25.8%)	<0.001
Business/employment	82 (21.4%)	33 (16.6%)	52 (23.1%)	
Visiting friends/relatives (VFR)	99 (25.8%)^**‡**^	26 (13.1%)	68 (30.2%)	
Other/not stated	33 (8.6%)	21 (10.6%)	47 (20.9%)	
**Duration of travel**				
<2 weeks	148 (38.5%)	36 (18.1%)	79 (35.1%)	<0.001
2 weeks - <3 months	183 (47.7%)	98 (49.2%)	56 (24.9%)	
3 months or more	48 (12.5%)	64 (32.2%)	87 (38.7%)	
**Travel group**				
Traveling alone	200 (52.1%)	92 (46.2%)	126 (56.0%)	0.06
Traveling with children (<16 years)	29 (7.6%)	29 (14.6%)	14 (6.2%)	0.01
**Visiting two or more countries**	138 (35.9%)	178 (89.4%)	90 (40.0%)	<0.001
**Destination**^¶^				
Australia	-	199 (100.0%)	225 (100%)	-
Cambodia	8 (2.2%)	7 (3.3%)	2 (1.0%)	
China	39 (10.0%)	14 (7.3%)	17 (7.5%)	
Hong Kong	60 (15.5%)	22 (11.1%)	16 (6.9%)	
Indonesia	21 (5.6%)	4 (2.0%)	3 (1.3%)	
Japan	4 (1.0%)	6 (3.2%)	6 (2.7%)	
South Korea	0 (0.0%)	1 (0.6%)	6 (2.7%)	
Laos	6 (1.5%)	3 (1.5%)	2 (1.0%)	
Malaysia	51 (13.2%)	10 (5.2%)	16 (6.9%)	
Philippines	13 (3.4%)	0 (0.0%)	6 (2.5%)	
Singapore	119 (31.1%)	81 (40.7%)	38 (17.0%)	
Thailand	90 (23.4%)	48 (23.9%)	18 (7.9%)	
Vietnam	16 (4.3%)	5 (2.4%)	5 (2.2%)	
India	5 (1.2%)	5 (2.4%)	9 (3.9%)	
Europe	80 (20.7%)	52 (26.2%)	7 (3.3%)	
New Zealand	4 (1.0%)	25 (12.5%)	4 (2.0%)	
Other	53 (13.8%)	52 (26.1%)	9 (3.9%)	

* Excludes travelers from other regions (n = 35).

† Other Western travelers includes residents of New Zealand, North America and Europe.

‡ 70.7% of Australian VFR travels were foreign-born Australians.

^¶^ Multiple destinations reported.

### **Pre-travel health-seeking**

At least one visit to a health professional in the past year for any reason was reported by 532/843 (63.1%) respondents, including 320 (38.0%) reporting two or more visits. Overall, 415 (49.2%) respondents sought some form of travel health information prior to their present trip. Pre-travel health advice was sought from a health professional by 298 (35.3%) respondents with 237 (79.5%) of these respondents seeking advice from their general practitioner. A travel specialist or travel clinic was attended by 35 (4.2%) respondents prior to travel or 11.7% of those who sought professional pre-travel health advice. Non-medical sources of travel health information were reported by 290 (34.4%) respondents. The Internet was reported as a source of pre-travel health information by 162 (19.2%) respondents, with 71/162 (43.8%) including a government travel advisory website in their web searches. Travel agents were reported as a source of travel health information by 114/843 (13.5%) respondents. Differences in pre-travel health seeking by region of residency are shown in Table 2. Seeking any pre-travel health information differed between study sites and was reported by 346 (47.5%) respondents departing Sydney and 69 (60.5%) respondents departing Bangkok (p = 0.009). No association was found between study sites in the proportions seeking professional health advice overall (p = 0.1), from a general practitioner (p = 0.3), travel specialist (p = 0.1) or a non-medical source (p = 0.1). Factors independently associated with seeking pre-travel health advice from a health professional on multivariate analysis differed by region of residence and are reported in Table 3.

**Table 2:** Reported pre-travel-health behaviors and vaccine uptake by traveler region of residence (N = 808*)

**Pre-travel health behavior**	**Region of residence**		
	**Australia** **(N = 384)**	**Other Western countries** **(N = 199)**	**Asia** **(N = 225)**
**Visited any doctor in the past year**	265 (69.0%)^a^	130 (65.3%) ^a^	117 (52.0%)
**Visited doctor for travel-health advice prior to this trip**			
Any health professional^†^	158 (41.1%) ^a^	76 (38.2%) ^a^	52 (23.1%)
General practitioner	129 (33.6%) ^a^	55 (27.6%)	46 (20.4%)
Travel doctor/clinic	13 (3.4%) ^b^	17 (8.5%) ^a^	5 (2.2%)
Other health professional	17 (4.4%)	7 (3.5%)	2 (0.9%)
**Sought travel health advice from non-medicalsource**			
Any non-medical source^†^	122 (31.8%)^b^	93 (46.7%) ^a^	62 (27.6%)
Travel agent	45 (11.7%) ^b^	36 (18.1%)	30 (13.3%)
Researched on the internet	46 (12.0%) ^a b^	43 (21.6%) ^a^	12 (5.3%)
Government travel advisory	28 (7.3%) ^b^	26 (13.1%)	17 (7.6%)
Other source	17 (4.4%)	11 (5.5%)	7 (3.1%)
**Source of travel health advice**			
None	181 (47.1%) ^a^	90 (45.2%) ^a^	140 (62.2%)
Health professional only	82 (21.4%)	16 (8.0%)	23 (10.2%)
Health professional/other source	76 (19.8%)	61 (30.7%)	28 (12.4%)
Other source only	45 (11.7%)	32 (16.1%)	34 (15.1%)
**Vaccinated**			
Any pre-travel vaccine^†^	46 (12.0%) ^a b^	43 (21.6%) ^a^	11 (4.9%)
Two or more vaccines	26 (6.8%) ^a b^	30 (15.1%) ^a^	5 (2.2%)
Hepatitis A	24 (6.3%)	30 (15.1%)	4 (1.8%)
Hepatitis B	19 (4.9%)	23 (11.6%)	4 (1.8%)
Tetanus	11 (2.9%)	16 (8.0%)	0 (0.0%)
Typhoid	17 (4.4%)	10 (5.0%)	2 (0.9%)
Influenza	16 (4.2%)	2 (1.0%)	6 (2.7%)
Measles/mumps/rubella	6 (1.6%)	2 (1.0%)	2 (0.9%)
Polio	7 (1.8%)	4 (2.0%)	0 (0.0%)
Cholera	5 (1.3%)	1 (0.5%)	1 (0.4%)

* Excludes travelers from other regions (n = 35).

† Multiple responses allowed.

^a^ % reporting outcome is significantly different to proportion reported by Asian travelers (p < 0.05).

^b^ % reporting outcome is significantly different to proportion reported by other Western travelers (p < 0.05).

**Table 3:** Independent predictors of seeking professional pre-travel health advice, by traveler region of residence

**Factor**	**Proportion seeking advice**	OR (95% CI)*	**P value**
**Australian travelers**			
**Age**			
16–35 years	73/185 (39.5%)	1.3 (0.8–2.4)	0.3
36–55 years	43/124 (34.7%)	1	
>55 years	41/74 (55.4%)	2.0 (1.0–3.9)	0.05
**Gender**			
Male	74/218 (33.9%)	1	
Female	83/165 (50.3%)	1.75 (1.1–2.9)	0.03
**Marital status**			
Married	98/205 (47.8%)	1	
Not married	54/163 (33.1%)	1.81 (1.1–3.0)	0.03
**Resident of birth country**			
No	55/169 (32.5%)	1	
Yes	100/212 (47.2%)	2.03 (1.3–3.3)	0.004
**Travel for business**			
No	145/314 (46.2%)	2.38 (1.1–5.0)	0.02
Yes	12/69 (17.4%)	1	
**Travel with others**			
No	112/292 (38.4%)	1	
Yes	46/92 (50.0%)	2.56 (1.6–4.2)	<0.001
**Other Western travelers**			
**Length of stay**			
<2 weeks,	7/35 (20.0%)	1	
2 weeks - <3 months	35/99 (35.4%)	1.80 (0.9–3.8)	0.1
3 months or more	34/64 (53.1%)	2.63 (1.3–5.4)	0.009
**Travel to South East Asia**			
No	16/68 (23.5%)	1	
Yes	60/130 (46.2%)	2.37 (1.2–4.7)	0.01
**Asian travelers**			
**Age**			
16–55 years	41/201 (20.4%)	1	
>55 years	10/23 (43.5%)	3.47 (1.3–9.0)	0.01
**Gender**			
Male	16/115 (13.9%)	1	
Female	36/111 (32.4%)	2.73 (1.4–5.4)	0.004
**Number of countries visiting**			
One	22/134 (16.4%)	1	
Two or more	29/90 (32.2%)	2.38 (1.2–4.6)	0.01

* Adjusted for other variables included in the logistic regression model.

### **Pre-travel vaccination**

Pre-travel vaccination rates were low across both study sites with 100 (11.9%) respondents reporting receipt of one or more vaccines specifically for this trip, including 73/298 (24.5%) of those reporting a pre-travel heath visit. Pre-travel vaccination differed by region of residence (Table 2). Travelers who reported seeking advice from a travel medicine specialist were more likely to report a pre-travel vaccine (21/36, 58.3%) compared to travelers reporting a pre-travel visit to a general practitioner (48/237, 20.3%, p < 0.001). The most commonly reported vaccines were hepatitis A vaccine (58, 6.9%), hepatitis B vaccine (46, 5.5%), typhoid vaccine (30, 3.6%), tetanus vaccine (28, 3.3%) and influenza vaccine (24, 2.8%). Other vaccines were reported by between 0.4% and 1.4% of respondents. Of those reporting a pre-travel vaccination, 40/102 (39.2%) recalled only one vaccine, predominantly influenza vaccine (16/40, 40.0%) or hepatitis A vaccine (10/40, 25.0%). Two pre-travel vaccines were recalled by 21/102 (20.6%) respondents and 41 (40.2%) reported three or more vaccines. Respondents departing Bangkok were more likely to report pre-travel vaccine uptake (21/114, 18.4%) than those departing Sydney (81/729, 11.1%, p = 0.03).

## **Discussion**

Seeking health-related information prior to travel may prepare travelers for health risks at their destination and studies have shown an association between receiving advice and accurate risk perception and undertaking preventative behaviors [4,5,16]. Considerable variation exists in the published literature on the proportion of surveyed travelers seeking pre-travel health advice, ranging from as low as 32% of departing travelers from Australasian airports [10] to 85-94% of those traveling to sub-Saharan Africa and Central and South America [7,17-19]. In our study, less than half of all respondents (49%) reported seeking health information from any source prior to travel, similar to a number of published studies [6-8,10,20]. We found travel to tropical regions, such as countries in South East Asia associated with higher rates of health seeking compared to travel to more temperate regions such as countries in North East Asia. The risk of infectious disease is not limited to low resource countries and travelers to Australia may still be at risk of infectious diseases, particularly in the tropical regions [21]. Visitors to Australia should be aware of the risks of travel at any destination.

While numerous health resources are available to travelers, it is recommended that travelers seek advice from a health professional prior to international travel [3,4]. In our survey, approximately two thirds of respondents traveled without professional medical advice. General practice was the main source of professional pre-travel health advice, reflecting results from other airport surveys of travelers [5,6,8,10] and providing further weight to the importance of general practice in preventative travel medicine. Despite differences in health systems, the majority of travelers internationally seek advice from their general practitioner [8,20,22]. This is particularly so in Australia, where general practitioners play a central role in the delivery of primary and preventative health care [23,24]. While some studies report increased knowledge and accurate risk perception in travelers who consult travel medicine specialists [4,5], only 4.2% of travelers in our study (ranging from 2.2% to 8.5% depending on region of residence) attended a travel clinic prior to travel. The role of the general practitioner is under-valued in travel medicine research and few studies of travelers who consult general practice are available. With this key role in the health of travelers, general practice is challenged with the provision of accurate and tailored advice during consultations that are limited by time and resources.

Pre-travel health seeking is influenced by many factors including traveler demographic characteristics, reasons for travel and previous travel experience. Few studies investigate differences in health-seeking norms by nationality. Our study showed that uptake of pre-travel health advice from a health professional differed considerably by region of residence. Health seeking is likely to differ by nationality due to differences in health seeking practices, country-specific healthcare systems including national vaccination programs and travel health facilities as well as promotional activities undertaken by health departments and private travel medicine groups. While it is likely that pre-travel health practices and vaccine uptake differs by destination and prior travel experience, pre-travel health advice may still be warranted and provides the opportunity to vaccinate if required. In Australia, a number of recent cases of measles have been imported from developed countries, and high risk Australian travelers to Europe and North America during the northern hemisphere winter are advised to receive the influenza vaccine [25]. Furthermore, many travelers travel to multiple destinations and may not be aware of the individual risks. Alongside other studies, our study confirms the low uptake of pre-travel health advice and vaccination among Asian travelers. Another airport survey conducted in the region found only 26% of Asian travelers reported seeking pre-travel health advice compared to 63% of Western travelers [10], while a separate study reported 23.9% of South Korean travelers to India [26], a high risk destination for many infectious diseases [27], sought pre-travel health advice. Expansion of the Asian travel market has been forecast due to increasing wealth within the region and growth in intra-regional tourist arrivals [28]. As more people within the Asia-Pacific region can afford to travel, it is important to increase the uptake of pre-travel advice, particularly from medical sources. The limited number of specialist travel clinics in low resource countries may be a current barrier to uptake of pre-travel health advice and differences in health seeking behaviors and associated factors by region of residence may limit the generalisability of traveler studies, highlighting the growing need to better understand travelers from emerging travel markets.

Travel to visit friends and relatives (VFR) is an established risk factor for acquiring infectious diseases during travel [29] and for poor uptake of pre-travel health advice [8,30]. We did not identify a significant association between VFR travel and uptake of professional pre-travel health advice. However, we found migrant Australian travelers to be half as likely to seek pre-travel advice from a health professional and more likely to be traveling to visit friends and relatives than Australian-born travelers. Recent evidence also suggests that ethnicity, in addition to travel to visit friends and relatives, is an important indicator of infectious disease risk during travel [30]. Australian migrants who travel may be at a greater risk of infectious diseases than Australian-born travelers due to their lower uptake of pre-travel health advice, regardless of reason for travel.

We acknowledge some limitations to our study. A brief self-administered questionnaire design, although appropriate to maximize the response rate in high volume airport surveys, limits the amount of detail obtainable and is also subject to recall bias. Additional factors which have been found to be associated with uptake of pre-travel health advice, such as previous travel experience and economic barriers were not obtained. Due to strict security measures at Sydney Airport, we were not able to gain access beyond customs and conducted our interviews in the departures check-in area. This resulted in a lower response rate than those reported by other airport surveys conducted in the departure lounges [26,31,32] in which a response rates have been reported. However, our method allowed for the recruitment of passengers from a variety of carriers and flight times including weekends and evenings during the study period, providing a representative cross-section of departing passengers. We utilized a number of techniques to ensure a representative sample of travelers including a multistage sampling method of flight selection and random participant recruitment in which all passengers joining selected check-in queues had an equal probability of selection excluding those few who exceeded the recommended check-in time before departure. The use of simplified English and the provision of additional language versions of the questionnaire by bi-lingual interviewers aimed to reduce language barriers and subsequent selection biases of visitors to Australia from Asia.

A low proportion of participants in this study reported receipt of pre-travel vaccines (12%) with lower rates of pre-travel vaccination reported by Asian travelers compared to Western travelers. Wilder-Smith et al. also reported low uptake of pre-travel vaccines among Asian travelers, with 5% of Asian resident respondents reporting any pre-travel vaccination and low rates of self-reported prior vaccination against common vaccine-preventable diseases [10]. The findings of this airport study were similar to other traveler surveys in that the most commonly reported vaccines received prior to travel were hepatitis A, hepatitis B, tetanus and typhoid [2,8,10,33]. Influenza vaccine was reported by <3% of participants, a vaccine not often reported in other traveler surveys. It is likely that awareness of influenza as a travel-associated disease has increased for both providers and travelers with a post-pandemic survey of travel clinic attendees reported 13% uptake of seasonal influenza vaccine [34]. The proportion reporting a pre-travel vaccine after pre-travel attendance at a specialist travel medicine clinic was almost three times that reported at a general practice visit. Sample size limitations precluded a detailed analysis of factors associated with individual vaccine choices. However, the demographic and travel characteristics significantly associated with travel vaccination differed by residency group. A limitation to this study is that we did not collect data on prior vaccine uptake and are unable to determine the proportion of travelers who did not receive vaccines prior to this trip due to prior disease or vaccine-induced immunity. This airport study, like much research assessing vaccine uptake, relies on the self-reported history of previous vaccination and is subject to recall bias. As the time since vaccination is likely to influence recall, pre-travel vaccines may not be as vulnerable to recall bias as those vaccines received routinely, particularly childhood vaccines. Errors introduced by self-reported history include the misclassification of the vaccine received, particularly hepatitis A and B vaccine [35] and underestimation of the number of vaccines received. Other surveys have found that respondents report vaccines for non-vaccine preventable diseases such as hepatitis C and malaria, indicating a poor knowledge of their vaccination history [36] and other studies have shown wide differences between perceived vaccination status and vaccination certificates or blood antibody levels in travelers and other population groups [7,37-39]. Without serological testing it is difficult to ascertain if those who visited a medical professional prior to travel were up-to-date with their vaccinations, if they were vaccinated but could not recall the vaccines received or if their visits were a missed opportunity to vaccinate. Difficulties in assessing vaccination history for travel vaccines would be alleviated by the carriage of a vaccination record card for all vaccines received by travelers along with their passport and other travel documents, such as the requirement for entry and exit from yellow fever endemic regions.

Lack of time has been reported as a reason for not seeking pre-travel advice and vaccinations in other studies [6-8]. Other studies have reported that approximately one third of travelers seek advice for their trip within 2 weeks of departure [8,23,33]. Almost half of travelers departing from airports in Australasia reported planning their trip less than two weeks prior to departure [10]. This results in lower rates of pre-travel health seeking, particularly from a health professional as well as the reduced uptake of vaccines, incomplete vaccination schedules for vaccines requiring more than one dose and lack of adequate protection at the time of departure. Although the length of time between health seeking and departure was not assessed in this airport survey, 63% of respondents had visited a health professional at least once in the past year, providing an opportunity for health professionals to inquire about possible overseas trips during their consultation and improve the provision of pre-travel health advice, particularly for migrants and those traveling to visit friends and relatives.

## **Conclusions**

The few travelers seeking advice suggests that public health strategies aimed at travelers may be warranted. These would include increasing uptake of pre-travel medical advice, ensuring vaccines, both routine and those recommended for travel, are up to date as well as the promotion of affordable and accessible travel clinics in low resource countries as traveler numbers increase. Limited data from the Asia-Pacific region are available and few studies assess traveler preparedness in residents of low resource countries, despite the strong growth of the international travel market in Asia. There is variability in pre-travel health advice seeking and vaccine uptake by region of residence and in migrant travelers. The role of general practitioners in the provision of pre-travel health advice and traveler health promotion is central and opportunities for improvement exist. Considering the increasing volume of international travel, a greater integration of travel history and travel plans into general practice consultations would identify those who intend to travel but are not aware of the need for a pre-travel health consultation, particularly migrant travelers. Determining the most appropriate strategies for increasing pre-travel health preparation, particularly for vaccine preventable diseases in travelers is the next stage in advancing travel medicine research.

## **Competing interests**

CRM receives funding from GSK and CSL Biotherapies for investigator-driven research. These payments were not associated with this study. The remaining authors have no competing interests.

## **Authors’ contributions**

CRM and RW conceived and supervised the study. AH organized airport access in Australia, undertook data collection and analysis and drafted the manuscript. SI and KN organized airport access in Thailand and undertook data collection. CRM, RW, SI and KN reviewed the manuscript. Selected results appearing in this manuscript were presented at the Asia-Pacific International Conference on Travel Medicine, Melbourne, Australia, 24–28 February 2008 and the 13th International Congress on Infectious Diseases, Kuala Lumpur, Malaysia, June 19–22, 2008. All authors read and approved the final manuscript.

## Pre-publication history

The pre-publication history for this paper can be accessed here:

http://www.biomedcentral.com/1471-2458/12/321/prepub
